# Influence of nozzle configurations on radiobiological effects in carbon ion radiation therapy estimated using GATE/Geant4

**DOI:** 10.1016/j.zemedi.2025.04.004

**Published:** 2025-05-08

**Authors:** Hermann Fuchs, Alessio Parisi, Keith M. Furutani, Dietmar Georg, Chris J. Beltran

**Affiliations:** a Department of Radiation Oncology, Mayo Clinic Florida, Jacksonville, FL, United States of America; b Department of Radiation Oncology, Medical University of Vienna, Vienna, Austria

**Keywords:** Carbon ion radiation therapy, Nozzle, Relative biological effectiveness, Monte Carlo, Microdosimetry

## Abstract

**Background:**

Carbon Ion Radiation Therapy (CIRT) has been used in Europe and in Asia for several decades. The first treatment facility in America is now under construction at Mayo Clinic Florida (MCF) in Jacksonville. CIRT is known to have a variable relative biological effectiveness (RBE) which depends on the microdosimetric spectra and consequently the kinetic energy spectra.

**Purpose:**

CIRT centers from different vendors exist around the world with different accelerators, delivery, and nozzle designs. Although nominally they provide comparable energies and beam qualities, this study investigates how the different nozzle designs might affect radiation quality and the consequent RBE.

**Methods:**

The impact of three nozzle designs, the upcoming MCF (Jacksonville, USA), MedAustron Ion Therapy Center (Wiener Neustadt, Austria), and the Osaka HIMAK (Osaka, Japan), on the RBE was investigated using OpenGATE10. The microdosimetric spectra were determined using the abridged microdosimetric distribution methodology (AMDM) and input into the MCF microdosimetric kinetic model (MKM) to determine spectral and RBE dependence on nozzle design. Monoenergetic carbon ion beams having a range in water of 3 and 27 cm were simulated. For the 27 cm beams, a simulated water-based range shifter was inserted before the phantom, reducing the range to 3 cm. Furthermore, a shallow spread-out Bragg peak (SOBP) (5-10 cm) and a deep SOBP (15-21 cm), were simulated for all nozzles and the resulting integrated dose profiles compared.

**Results:**

For all nozzle geometries, the range at 80% dose fall-off (R80) agreed within 0.1 mm. The lineal energy and the RBE agreed very well until the Bragg peak, after which some differences could be observed. For the SOBPs, the agreement was similar with an agreement in the biological dose before and at the SOBP within 0.7%. For the thick nozzle of Osaka HIMAK, small differences were observed, mostly in the fragmentation tail.

**Conclusion:**

The AMDM was successfully integrated into OpenGATE10 and used to compute the RBE with the MCF MKM. It was shown that the nozzle design itself had only a minor effect on the radiation quality and consequently the RBE. A small difference in RBE is observed mostly after the Bragg peak and SOBP in the fragmentation tail and depends on the nozzle water equivalent tissue (WET), when it is a change of more than 24 mm.

## Introduction

Due to their physical characteristics, carbon ion radiation therapy (CIRT) allows for improved dose conformity compared to conventional photon-based irradiation. In addition to the physical dose characteristics, CIRT is generally characterized by a higher relative biological effectiveness (RBE) with respect to the X-rays used in conventional radiation therapy.

In proton therapy, the RBE used for clinical purposes is assumed to be constant at a fixed 1.1. For carbon ions it is variable, and several models have been used [1] such as the local-effect model (LEM 1) and the modified microdosimetric-kinetic model (modified MKM) [2,3]. The models have their strengths and weaknesses [4], [5], [6], [7], [8]. The Mayo Clinic Florida microdosimetric kinetic model (MCF MKM) [9] aims to be an improvement on the existing modified MKM. The MCF MKM predicts the in-vitro clonogenic survival in the low as well as the high linear energy transfer (LET) range using computationally efficient single-event microdosimetric spectra [9].

Our group recently introduced a methodology, named abridged microdosimetric distribution methodology (AMDM), enabling a much faster and more efficient generation of the voxel-by-voxel microdosimetric spectra required for the RBE calculations [10].

CIRT centers from different vendors are in operation all over the world. Recently, the Mayo Clinic Florida (MCF) announced the construction of a synchrotron-based ion-therapy center being able to provide proton and carbon ion for treatment [11]. All CIRT centers provide nominally comparable carbon ion beams in terms of energy or spot size. However, due to different designs of the beam delivery system and the respective nozzles, it is not fully clear whether the different materials involved might alter the radiation quality.

This article focuses on what extent the possible changes in the microdosimetric spectra due to nozzle design could affect the RBE. To this aim, we have modelled the treatment nozzles of three different CIRT centers, Mayo Clinic Florida (Jacksonville, Florida, USA), MedAustron ion therapy center (Wiener Neustadt, Austria), and the Osaka Heavy Ion Therapy Center (HIMAK, Osaka, Japan), using the open-source Monte Carlo code OpenGATE [12]. To facilitate comparisons, the AMDM [10] was implemented into OpenGATE to compute the voxel-specific microdosimetric spectra and those spectra then used to determine the RBE with MCF MKM model. The biological impact of the various nozzle designs was then compared.

## Materials & methods

### Monte Carlo tool kit GATE

The development version of OpenGATE10, based upon the Monte Carlo toolkit Geant4 v11.2.1 was employed for this study[12]. OpenGATE10 is designed to lower the user threshold for Monte Carlo simulations as much as possible. It employs a python wrapper, allowing user interaction directly from python scripts, without having to refer to C++ or macro commands. Installation can be performed using a simple pip install command without requiring the user to manually download and compile multiple software packages. OpenGATE10 is an open-source toolkit for Monte Carlo simulations initially developed for nuclear imaging applications but later extended towards other modalities such as CIRT [12], [13], [14], [15], [16].

### AMDM implementation

The AMDM [10] was implemented into OpenGATE10 by the authors as a so-called actor, allowing to store spatially resolved multi-dimensional data in user defined resolutions.

**Fig. 1:**
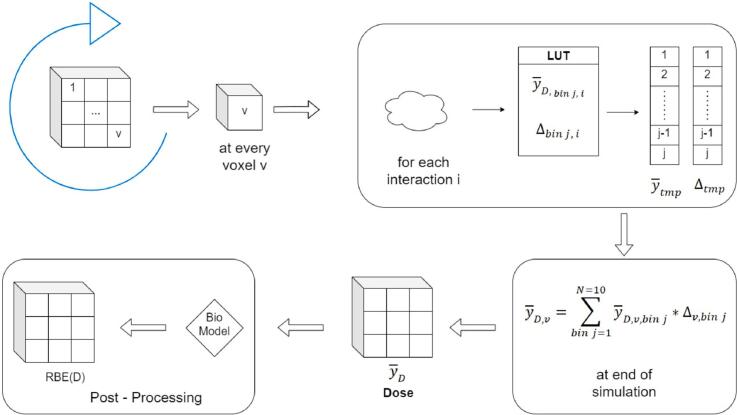
A graphical overview of the implemented OpenGATE AMDM actor and the post processing

The AMDM was presented in detail elsewhere [10]. Here only a short summary is given. A graphical overview of the implemented process can be found in Fig. 1.

The distribution of dose mean lineal energy was discretized to lineal energy intervals (bins), for memory efficiency. This resulted in the microdosimetric quantity dose mean lineal energy ${\bar{y}}_{{D,}_{\mathrm{bin}\;j}}$ and the energy deposited per interaction $\Delta_{\mathrm{bin}\;j}$ being calculated over a given energy range and stored for each individual voxel during the calculation.

In order to be computationally efficient, the bin-dependent values of the dose mean lineal energy ${\bar{y}}_{{D,}_{\mathrm{bin}\;j}}$ as well as of the energy deposited per interaction $\Delta_{\mathrm{bin}\;j}$ were pre-calculated using the most recent version of the analytical microdosimetric function [17] implemented in PHITS [18] and stored in a look-up table (LUT) based on particle type and energy. These two quantities were calculated with PHITS as in Equations (1), (2)

(1) $${\bar{y}}_{{D,}_{\mathrm{bin}\;j}}=\frac{\sum_{y_{\mathrm{bin}\;j,min}}^{y_{\mathrm{bin}\;j,max}}yd\left(y\right)dy}{\sum_{y_{\mathrm{bin}\;j,min}}^{y_{\mathrm{bin}\;j,max}}d\left(y\right)dy}$$

(2) $$\Delta_{\mathrm{bin}\;j}=\sum_{y_{\mathrm{bin}\;j,min}}^{y_{\mathrm{bin}\;j,max}}d\left(y\right)dy$$

where *d(y)* is the dose probability density distribution of the lineal energy *y*.

The look-up table was generated for ions from hydrogen to neon ions. For each ion, the look-up table includes 400 logarithmically spaced kinetic energy values between 1 MeV/u to 1 GeV/n. Isotopes were treated like the main stable ion. For particle energies not contained in the LUT the values were linear interpolated. Particles not covered in the LUT were not considered for the calculation. Data of the corresponding bins was stored in corresponding dimensions. The number of lineal energy bins *j* was fixed to 10 in order to balance accuracy and memory consumption. The energy intervals were chosen heuristically with 5 bins until 100 keV and the remaining bins covering the energy range up to 1000 keV. The LUT were calculated using PHITS.

During the OpenGATE simulations, at each voxel *v*, for every interaction *i*, ${\bar{y}}_{D,bin\;j}$ and $\Delta_{\mathrm{bin}\;j}$ are extracted from the look-up tables and added to their current values in the voxel as a dose-weighted sum. In addition, the macroscopic energy $e_{v,i}$ deposited in the voxel *v* at every interaction *i* is stored resulting in $E_{v}=\sum e_{v,i}$. For each voxel, three output files were generated containing ${\bar{y}}_{{D,}_{\mathrm{bin}\;j}}$, $\Delta_{\mathrm{bin}\;j}$, and $E_{v}$.

Finally, these AMDM quantities were used to compute the RBE, as detailed in the following paragraph.

### RBE calculations with the MCF MKM

The voxel-specific values of the linear (α) and quadratic (β) terms of the linear-quadratic model of cell survival [20] were calculated using the AMDM-implementation [10] of the MCF MKM [9]. The RBE calculations were carried out for the clonogenic survival of human salivary gland tumor cells (HSG cell line).

$$\alpha_{\mathrm{bin}\;j}=\left(\alpha_{0}+{0.1602\beta }_{0}\frac{{\bar{y}}_{D_{\mathrm{bin}\;j}}}{\rho \pi r_{d}^{2}}\right)c_{bin\;j}$$

$$\alpha =\sum_{j}\alpha_{\mathrm{bin}\;j}\Delta_{\mathrm{bin}\;j}$$

$$c_{\mathrm{bin}\;j}=\frac{1-exp\left[-\left(\alpha_{0}+\beta_{0}0.1602\frac{{\bar{y}}_{D_{\mathrm{bin}\;j}}}{\rho \pi r_{d}^{2}}\right)0.1602\frac{{\bar{y}}_{D_{\mathrm{bin}\;j}}}{\rho \pi R_{n}^{2}}-\beta_{0}{\left(0.1602\frac{{\bar{y}}_{D_{\mathrm{bin}\;j}}}{\rho \pi R_{n}^{2}}\right)}^{2}\right]}{\left(\alpha_{0}+\beta_{0}0.1602\frac{{\bar{y}}_{D_{\mathrm{bin}\;j}}}{\rho \pi r_{d}^{2}}\right)0.1602\frac{{\bar{y}}_{D_{\mathrm{bin}\;j}}}{\rho \pi R_{n}^{2}}+\beta_{0}{\left(0.1602\frac{{\bar{y}}_{D_{\mathrm{bin}\;j}}}{\rho \pi R_{n}^{2}}\right)}^{2}}$$

$$c=\sum_{j}c_{\mathrm{bin}\;j}\Delta_{\mathrm{bin}\;j}$$

$$\beta =\beta_{0}c^{2}$$

Afterwards the dose-dependent RBE was calculated as:

$$\mathrm{RB}E_{D}=\frac{\sqrt{\alpha_{\mathrm{ref}}^{2}+4\beta_{ref}\left(\alpha D+\beta D^{2}\right)}-\alpha_{\mathrm{ref}}}{2\beta_{ref}D}$$

Parameters used in the MCF MKM calculations are listed in Table 1.

**Table 1:** Parameters used for RBE calculations using the MCF MKM.

Parameter	Value
Cell line	HSG
*r*_d_	0.28 µm
*R_n_*	4.5 µm
α_0_	0.117 Gy^-1^
β_0_	0.0615 Gy^-2^
$\alpha_{\mathrm{ref},\;6MV\;X-rays}$	0.217 Gy^-1^
$\beta_{\mathrm{ref},\;6MV\;X-rays}$	0.0615 Gy^-2^

#### Validation

Validation of the implementation as well as the used Monte Carlo engine Geant4 was performed by a comparison with reference simulations in water conducted using PHITS v3.33 [18] for a 330 MeV/u monoenergetic carbon ion beam. In these simulations, the entire microdosimetric spectrum was simulated based on the analytical microdosimetric function implemented in PHITS [19]. Physical dose, dose mean lineal energy as well as the RBE were compared.

### Nozzle and beam modelling

#### MedAustron

The clinical nozzle of the MedAustron ion therapy center (MedAustron iontherapy center, Wr. Neustadt, Austria) has been modelled previously [21], [22], [23], [24]. Although details are subject to non-disclosure agreements with the manufacturer, the overall structure is as follows. The nozzle consists of a double foil vacuum window, three monitoring chambers (the Independent Termination System and two Dose Delivery System chambers), two PMMA Ripple Filters and a 3 cm PMMA range shifter. The passive elements can be remotely configured to be inside the beam path. For carbon ions, both ripple filters are employed simultaneously and feature a triangular shape [23]. The monitoring chambers contain integrating as well as strip chambers. These consist of Mylar and Kapton foils coated with Aluminum. The total water equivalent thickness (WET) of the nozzle was evaluated to be 2.4 mm, with the nozzle exit window located 65 cm upstream of the isocenter. During modeling the virtual accelerator parameters before the nozzle were tuned such that the beam best matched the commissioning data. The particles leaving the treatment nozzle were stored into separate phase space files for each mono energetic energy. These phase space files were then used as particle sources for all simulations in this manuscript.

#### Mayo Clinic Florida (MCF)

For the MCF treatment nozzle (Mayo Clinic, Jacksonville, Florida, USA), confidential proprietary detailed manufacturer data sheets were used to model the nozzle components in OpenGATE10 directly. Similarly to the MedAustron nozzle three monitoring chambers are used. For carbon ions, a ripple filter composed of Acrylnitril-Butadien-Styrol (ABS) is available, featuring a novel design which was implemented based on CAD drawings. The fixed carbon ion beam line features an extensible nozzle allowing to adjust the distance between the nozzle exit and the isocenter in the range from 20 to 120 cm. For simplicity only the fully retracted nozzle was used in this manuscript. As the MCF facility is currently under construction, no existing beam data was yet available. Instead, the beam parameters were modelled based on design specifications and manufacturer data. The total WET of the nozzle (excluding ripple filter) was evaluated to be 1.6 mm. To allow a direct comparison, the ranges of the MCF system were matched with the MedAustron ranges in terms of the range at 80% dose fall off (R80) in order to create a beam model.

#### Osaka HIMAK

The nozzle of the Osaka Heavy Ion Therapy Center (Osaka HIMAK, Osaka, Japan) [25], [26], coming from the same vendor as the MCF nozzle, has some similarities to the MCF treatment nozzle, but features a different energy selection system using dynamic Range Shifters (RS), resulting in a substantially different WET. To mimic the low energy beam of the Osaka HIMAK, the MCF treatment nozzle described above was employed, using an additional 24 mm ABS RS located at the nozzle exit, closely resembling the actual HIMAK nozzle. Again, the ranges of the Osaka nozzle were matched in R80 to the MedAustron ranges for beam model generation.

### Simulation set-up and post processing

Simulations were performed using carbon ions with a development version of OpenGATE10 alongside Geant4 v11.2.1. The physics list Shielding with the new light ion QMD and the electromagnetics option4 with global production and stopping cuts of 10 cm and smaller cuts of 0.1 mm in the water phantom were employed. The light ion QMD model was implemented into Geant4 v11.2 and is supposed to improve light ion yields for more realistic particle beam therapy [27]. A total of primary 10^7^ carbon ions were simulated for each set-up, resulting in statistical uncertainties for all energies far below 1‰.

After exiting the nozzle (in case of the MCF and Osaka nozzle) or the corresponding phase space (for the MedAustron nozzle) the treatment beam impinged a water phantom with dimensions of 50x40x40 cm^3^ located such that the phantom entrance coincided with the isocenter. For every simulation, the output included the integrated physical dose (IDD) deposited in each voxel with a resolution of 0.1 mm. In addition, the multidimensional AMDM output was stored in three files (restricted energy deposition, dose-mean lineal energy histogram, fraction of absorbed dose deposited by lineal energy events histogram) having the same special resolution.

After the simulations were concluded, an in-house developed python script was employed calculating the RBE weighted dose employing the MCF MKM as described above, based on the parameters listed in Table 1. Furthermore, the carbon ion energy spectra at the beginning of the water phantom, and for the SOBP plans in addition at the center of the SOBP was evaluated.

### Nozzle comparison

The effects of different treatment nozzles on the resulting physical as well as biological dose distributions was investigated using mono energetic as well as more clinically relevant spread-out Bragg peaks (SOBPs).

#### Mono energetic

In a first step, mono energetic carbon ion beams with a range in water of 3 and 27 cm, corresponding to approximately 120 and 400 MeV/u were simulated, termed low and high energy in this manuscript, respectively. The low energy beam impacted the water phantom directly.

The high energy beam passed through an additional fictious RS, placed directly at nozzle exit, consisting solely of water before impinging the water phantom. The thickness of the RS was chosen such that the R80 of the higher energy was reduced to match the 3 cm range in R80 of the lower energetic beams. The same air gap as for the low energy set-up was achieved by moving the water phantom backwards in beam direction. Due to different fragmentation behavior, evaluation was focused before the Bragg peak (until R80).

#### Spread-out Bragg peaks (SOBPs)

In a second step, using the same set-up, two biologically optimized SOBPs of different depths were simulated. A shallow SOBP from 5 to 10 cm, and a deep SOBP ranging from 15 to 21 cm, both having a prescription dose of 3 Gy[RBE].

Biological optimization was performed using an in-house developed python script employing look-up tables for the physical dose as well as α and β, calculated using AMDM and MCF MKM for mono energetic beams. Biological plan optimization was performed only for the MCF nozzle. The same treatment plans with the same weights were used subsequently for all nozzles, e.g. no dedicated optimization was performed for the MedAustron or Osaka nozzle. This was purposely done in order to highlight potential differences.

## Results

### Validation of the AMDM implementation in OpenGATE

**Fig. 2:**
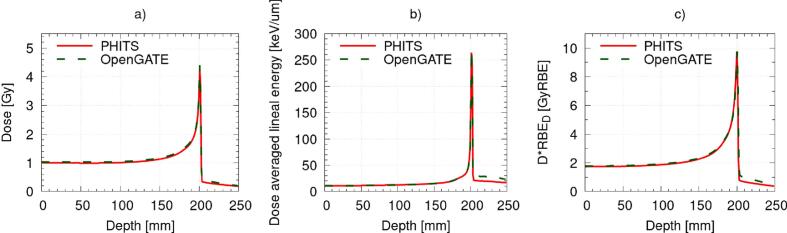
Comparison of a) physical dose, b) lineal energy, and c) RBE for a monoenergetic 330 MeV/u carbon ion beam in water simulation data using PHITS and the OpenGATE10 implementation of AMDM.

A comparison of the monoenergetic carbon ion beam in water between PHITS and the AMDM implementation in OpenGATE can be found in Fig. 2. The particle ranges agreed within 0.1 mm. The lineal energy agreed very well until the Bragg peak, with slight deviations after the Bragg peak. A similar effect was seen for RBE, where after the Bragg peak a notable difference could be observed.

### Nozzle comparison

#### Mono energetic

**Fig. 3:**
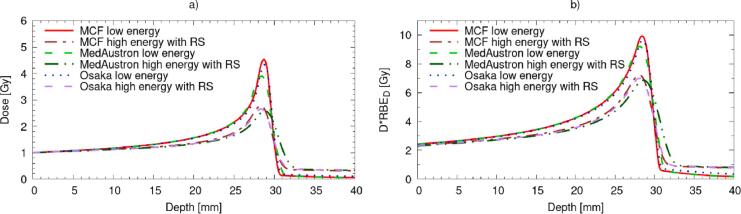
a) IDD curves and b) RBE weighted dose of carbon ions for the MCF, Osaka, and MedAustron nozzles. Range shifters were introduced to match the ranges to 3 cm for all initial energies and beam lines. The absorbed dose profiles (a) were normalized to 1 Gy at the entrance of the phantom.

A comparison of IDD curves for all nozzle configurations is displayed in Fig. 3a. The range at 80% dose fall-off was matched to be within 0.1 mm between all nozzle configurations and beams with and without RS, respectively. As expected, the Bragg-peak width is increased for the initially higher energetic particle beams due to range straggling [28].

Fig. 3b shows the RBE-weighted dose profiles for the low and high energetic beams which match well between the respective nozzle configurations. Only for the Osaka nozzle, differences in the fragmentation tail behind the Bragg peak can be observed compared to the MCF and MedAustron nozzle. As expected from the broadening of the Bragg peak itself [28], the RBE of the broader Bragg peaks of the high energy carbon ions including a RS was up to 10.7%, 13.5%, and 12.0% lower than the low energy beams, for the MCF, MedAustron, and Osaka nozzle, respectively. The maximum RBE for the low energy beams was 9.9, 9.2, and 9.6 for the MCF, MedAustron and Osaka nozzle, while 7.2, 6.9, and 7.0 for the MCF, MedAustron and Osaka high energy beams, respectively. The average RBE of all nozzles until the Bragg peak agreed within 9.5%.

**Fig. 4:**
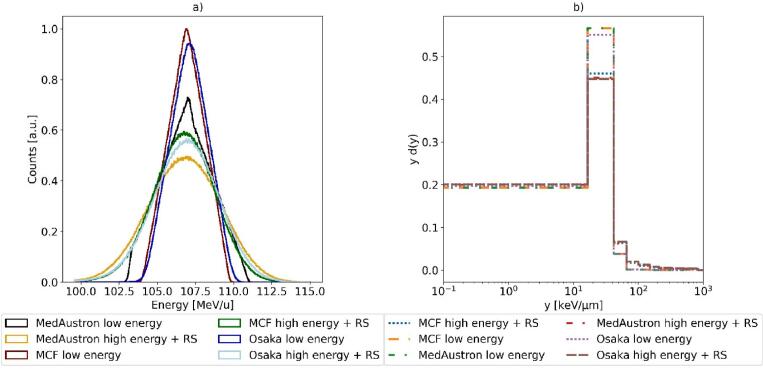
a) Energy spectra at the water phantom entrance for all investigated configurations. Data was normalized to the mean energy of the MedAustron low energy beam, b) Microdosimetric spectra as calculated using AMDM scored at the phantom entrance for the monoenergetic carbon ion beams.

A comparison of the energy spectra (see Fig. 4a) showed that the overall mean energies agreed within 0.2 MeV/u. Due to energy straggling the width of the energy spread was increased for the initially higher energetic particle beams, compared to the initially low energetic beams. The energy distribution of the respective nozzle configurations notably differs. This is most likely due to the different initial energy spread as well as the ripple filters used for broadening of the Bragg peak but as can be seen in Fig. 3 these differences manifest only marginal differences in Bragg peak shape. The microdosimetric spectra calculated using AMDM are displayed in Fig. 4b.

#### Spread-out Bragg peaks (SOBPs)

**Fig. 5:**
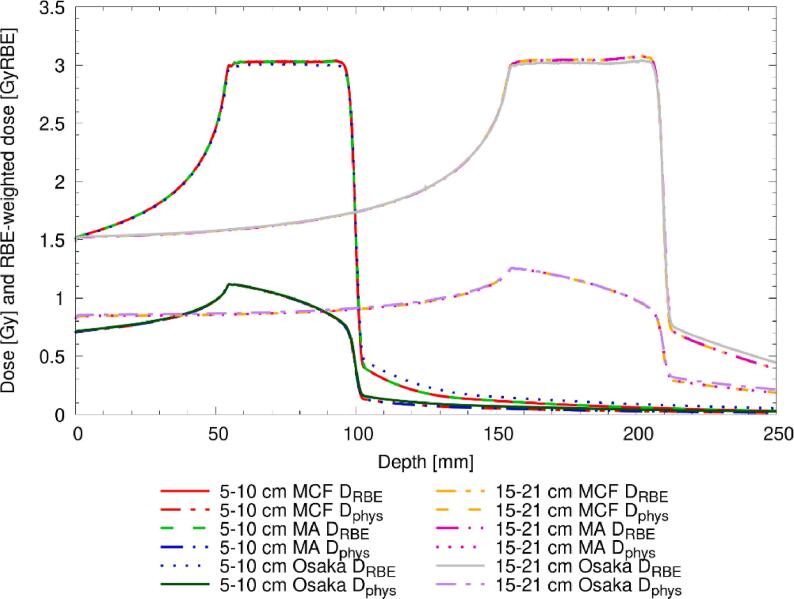
IDD curves of a shallow and deep carbon ion SOBP for the MCF, Osaka, and MedAustron nozzles. Both physical and biological weighted dose are displayed.

**Fig. 6:**
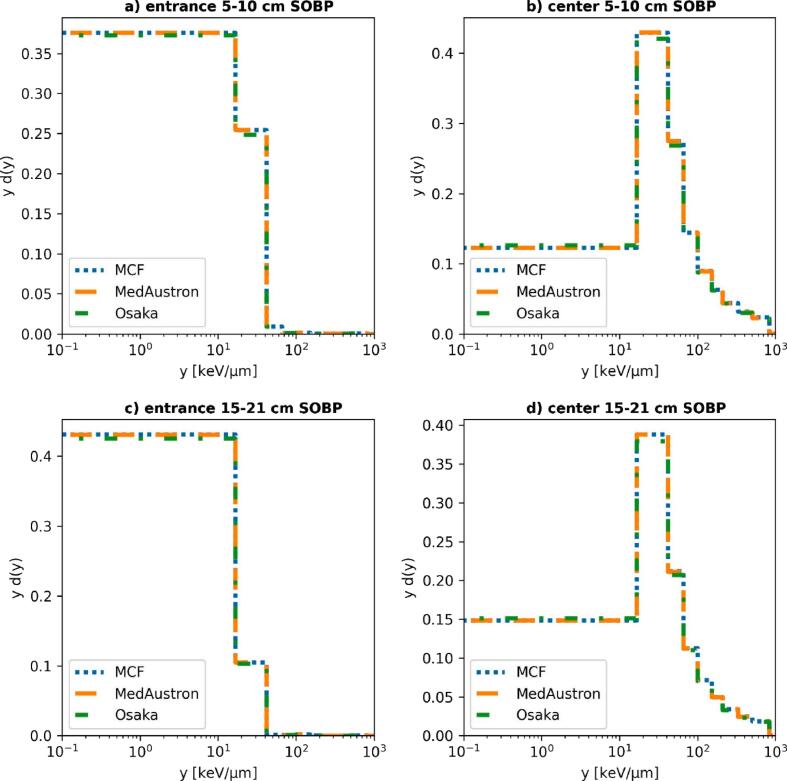
Microdosimetric spectra as calculated using AMDM scored at a) the phantom entrance for the 5-10 cm SOBP, b) center of the 5-10 cm SOBP, c) phantom entrance for the 15-21 cm SOBP, and d) center of the 15-21 cm SOBP.

A comparison of physical as well as biologically weighted dose for the shallow as well as the deep SOBP for all nozzle configurations can be found in Fig. 5 and Table 2. The physical dose profiles match very well for all SOBPs and nozzle configurations with an average agreement within 1% until the fragmentation tail. For the biological dose, R80 agrees within 0.1 mm across all SOBPs and nozzle configurations. The microdosimetric spectra calculated using AMDM is displayed in Fig. 6.

**Table 2:** Comparison of physical and biological dose parameters for the shallow and deep SOBP for the MCF, MedAustron and Osaka nozzle.

Nozzle	SOBP	ROI	Phys. Dose [Gy] (STDEV)	biol. Dose [Gy(RBE)] (STDEV)	Difference biol. Dose to MCF [%]
MCF	5-10 cm	Before SOBP	0.8 (0.0)	1.7 (0.1)	-
		SOBP	1.0 (0.09)	3.0 (0.01)	-
		Fall-off	0.0 (0.0)	0.1 (0.1)	-
	15-21 cm	Before SOBP	0.9 (0.0)	1.7 (0.2)	-
		SOBP	1.2 (0.07)	3.0 (0.01)	-
		Fall-off	0.2 (0.0)	0.5 (0.1)	-
MedAustron	5-10 cm	Before SOBP	0.8 (0.0)	1.7 (0.1)	0.02
		SOBP	1.0 (0.09)	3.0 (0.01)	0.003
		Fall-off	0.0 (0.0)	0.1 (0.1)	0.7
	15-21 cm	Before SOBP	0.9 (0.0)	1.7 (0.2)	-0.01
		SOBP	1.2 (0.07)	3.0 (0.01)	-0.03
		Fall-off	0.2 (0.0)	0.5 (0.1)	0.3
Osaka	5-10 cm	Before SOBP	0.8 (0.0)	1.7 (0.1)	-0.3
		SOBP	1.0 (0.09)	3.0 (0.01)	-0.9
		Fall-off	0.1 (0.0)	0.1 (0.1)	44.1
	15-21 cm	Before SOBP	0.9 (0.0)	1.7 (0.2)	0.2
		SOBP	1.2 (0.07)	3.0 (0.01)	-0.9
		Fall-off	0.3 (0.0)	0.6 (0.1)	10.3

For the shallow SOBP from 5 to 10 cm, the physical dose of the MCF nozzle agreed on average within 0.03% and 0.3% for the MedAustron and the Osaka nozzle, respectively. The biological dose before the SOBP differed from the MCF nozzle on average by 0.02% for the MedAustron and -0.3% for the Osaka nozzle. Within the SOBP the MedAustron nozzle agreed on average with 0.01% and the Osaka nozzle within -0.9%. In the fragmentation tail, after the SOBP larger deviations were observed of 0.7% and 44.1% for the MedAustron and Osaka nozzle, respectively.

For the deeper SOBP ranging from 15 to 21 cm, similar results were found. The physical dose in the SOBP of the MCF nozzle agreed on average within 0.1% and 0.2% for the MedAustron and the Osaka nozzle, respectively.

The biological dose before the SOBP of the MCF nozzle agreed within -0.01% and 0.2%, in the SOBP within -0.03% and -0.9% for the MedAustron and Osaka nozzle, respectively.

For both SOBPs, the Osaka nozzle shows slightly higher physical as well as biological doses in the fragmentation tail compared to the other nozzles due to change in fragmentation caused by the 24 mm ABS RS. The biologically weighted dose for the MCF and MedAustron nozzle agree very well. Again, the Osaka nozzle shows larger differences with slightly less biological dose in the SOBP while having more biological dose in the fragmentation tail.

**Fig. 7:**
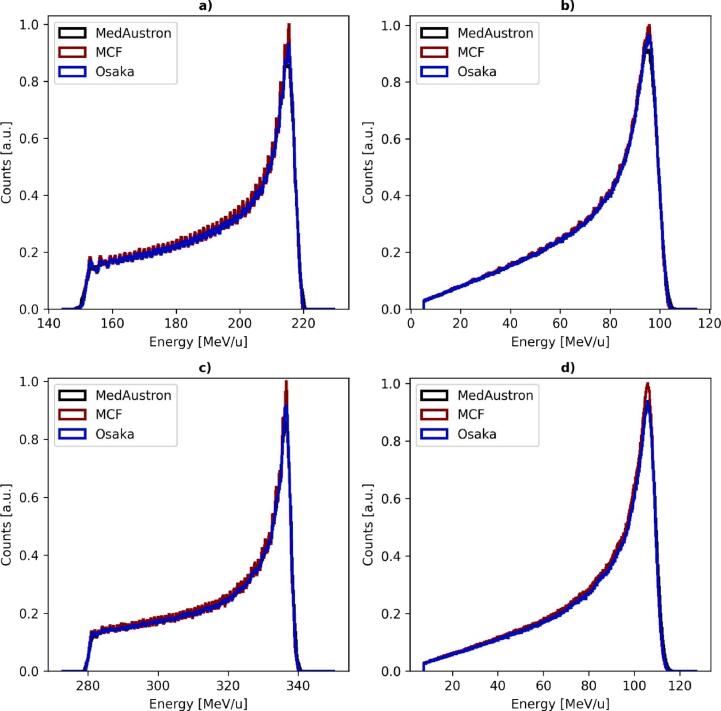
Energy spectra of carbon ions at the a) phantom entrance for the 5-10 cm SOBP, b) center of the 5-10 cm SOBP, c) phantom entrance for the 15-21 cm SOBP, and d) center of the 15-21 cm SOBP

The energy distribution of the primary carbon ions at the beginning of the water phantom and the center of the shallow and deep SOBP, respectively, are shown in Fig. 7. The mean energy agrees within 1 MeV/u. At the phantom entrance, a wobbling sub-structure can be seen for the MCF and Osaka nozzle, which is due to a different initial energy spread as well as ripple filter design. However, this does not seem to result in considerable changes in dose or biological dose distribution (see Fig. 3, and Fig. 5).

## Discussion

The OpenGATE implementation of AMDM resulted in similar data as the reference values generated with PHITS and indicate that the AMDM implementation can adequately calculate biological effects within the uncertainties of current physics fragmentation models. The prominent shapes and peak structures were well reproduced. Notable differences were found only after the Bragg peak. Unfortunately, it is not directly possible to attribute this to differences in physics modelling between Monte Carlo codes. A cursory investigation with different Geant4 versions as well as physics models also show differences between versions. The same might understandably be true for different PHITS versions or nuclear models implemented in that code. Noteworthy, the new physics list option light ion QMD only had a limited impact compared to the standard QMD implementation. However, as we only investigated IDD curves, more pronounced effects may be possible for other scenarios.

The overall shapes of the mono energetic Bragg peaks for the MCF, Osaka HIMAK, and MedAustron nozzles were found to be very similar. Differences between the peak heights are most likely related to the different specifications of the ripple filters. This was substantiated by the energy spectra. For the dose contribution a sensitivity study indicated that a variation in the initial energy spread before the nozzle from 0.01% to 0.1% had only a negligible effect on the physical dose distribution, despite being visible in the energy spectra themselves.

The PHITS simulations upon which AMDM was based included the whole lineal energy spectrum and were validated against experimental spectra previously [19]. The GATE AMDM simulations agreed well with the PHITS simulations (see Fig. 2), validating the approach.

Beam modelling of the MedAustron nozzle used a well-established method. The initial virtual beam parameters before the nozzle were tuned to best match experimental measurements. These measurements were based on measurements in air and water to determine energy, energy spread as well as spot size, which are also used for treatment planning system commissioning. Tuning of the MCF and Osaka nozzle was performed in a similar manner. As can be seen in Fig. 4, the low energy spectra for the MedAustron nozzle differs notably. Most likely this is due to a different geometry of the employed ripple filters. No microdosimetric data was used during beam modelling. Consequently, it is possible that features, such as fragmentation cross-sections or particle spectra, not strongly dependent on these factors are not perfectly modelled. This could be addressed using more detailed measurements involving energy spectra or beam quality data.

For the monoenergetic beams, range straggling resulted in a wider Bragg peak width for the carbon ion beams transversing the water range shifters compared to the lower energetic carbon ions (see Fig. 3). This was also evident in the kinetic energy spectra (see Fig. 4). The resulting RBE curves were similar with a few notable changes. A change in RBE after the Bragg peak was present for the beams traversing the range shifter, indicating a small change in beam quality. The SOBP for the different nozzle geometries matches closely. Like the monoenergetic cases, small differences for the biological dose were present in the fragmentation tail of the SOBP, but less pronounced due to the combination of multiple energy layers.

In a second step, using look-up tables calculated using the AMDM, biological optimized SOBPs were generated. As these were only optimized for the MCF nozzle, differences in biological effectiveness due to nozzle geometry or materials should therefore be much more pronounced. The absence of large effects further underlines the limited change of RBE due to similar though not identical nozzle design.

The MCF and MedAustron nozzle had a WET in the range of 1-3 mm. The Osaka nozzle on the other hand, due to the range shifter, has a WET in the order of 3-4 times larger. Observed changes in physical and more obviously in biological dose distributions after the SOBP for this nozzle seem to indicate that the material in the nozzle can in fact influence the beam quality. Although the differences of up to 45% appear rather large, the absolute dose in comparison to the SOBP dose is rather low. Consequently, given the difference between nozzles was quite large, the resulting differences were relatively minor. The nozzle with the highest differences (Osaka) showed ∼1% difference in biological dose. The nozzle of the Heidelberg Ion Beam Therapy Center (HIT) [30] and the National Institutes for Quantum and Science and Technology (QST), formerly National Institute of Radiological Sciences (NIRS) [29] are published and are rather similar to the MCF and MedAustron nozzle. Consequently, it stands to reason that all these nozzles have similar kinetic energy spectra, microdosimetric spectra, and biological effectiveness.

## Conclusion

The AMDM was integrated into the open-source Monte Carlo toolkit OpenGATE10 and used to calculate the RBE with the MCF MKM in different scenarios to quantify the effect of different nozzles on the biological dose of carbon ions.

Our results indicate that the nozzle design itself had only a minor effect on the radiation quality and consequently the RBE with changes below 1% before and at the SOBP.

## Data sharing

The data is available from the authors on request.

## Novelty and Significance of Study

Carbon Ion Radiation Therapy (CIRT) has been used in Europe and in Asia for several decades and the first treatment facility in the USA is now under construction in Jacksonville, Florida with installation beginning this year. CIRT depends on the microdosimetric spectra which depends on the kinetic energy spectra. There has never been a report of the impact of the vendor nozzle designs and this is the first such report.

## CRediT authorship contribution statement

**Hermann Fuchs:** Writing – review & editing, Writing – original draft, Visualization, Validation, Software, Resources, Project administration, Methodology, Investigation, Formal analysis, Data curation, Conceptualization. **Alessio Parisi:** Writing – review & editing, Writing – original draft, Validation, Software, Methodology, Formal analysis, Conceptualization. **Keith M. Furutani:** Writing – review & editing, Writing – original draft, Supervision, Resources, Project administration, Funding acquisition, Conceptualization. **Dietmar Georg:** Supervision. **Chris J. Beltran:** Writing – review & editing, Supervision, Resources, Project administration.

## Declaration of competing interest

The authors declare that they have no known competing financial interests or personal relationships that could have appeared to influence the work reported in this paper.
